# Optimizing formaldehyde and binary ethylenimine combinations for inactivation of foot-and-mouth disease virus GR12: insights from Indonesia’s third outbreak for local vaccine development

**DOI:** 10.14202/vetworld.2025.2798-2810

**Published:** 2025-09-23

**Authors:** Firdausy Kurnia Maulana, Nur Saidah Said, Zayyin Dinana, Deka Uli Fahrodi, Jola Rahmahani, Yulianna Puspitasari, Suryo Kuncorojakti, Helen Susilowati, Diyantoro Diyantoro, Maryono Maryono, Fedik Abdul Rantam

**Affiliations:** 1 Doctoral Program in Veterinary Science, Faculty of Veterinary Medicine, Universitas Airlangga, Surabaya, East Java, Indonesia; 2 Airlangga Disease Prevention and Research Center, Universitas Airlangga, Surabaya, East Java, Indonesia; 3 Division of Veterinary Microbiology, Faculty of Veterinary Medicine, Universitas Airlangga, Surabaya, East Java, Indonesia; 4 Research Centre for Vaccine Technology and Development, Institute of Tropical Disease, Universitas Airlangga, Surabaya, East Java, Indonesia; 5 Department of Animal Husbandry, Faculty of Animal Husbandry and Fisheries, Universitas Sulawesi Barat, Majene, Indonesia; 6 Department of Viral Diarrhea, Institute of Tropical Disease, Universitas Airlangga, Surabaya, Indonesia; 7 Division of Veterinary Anatomy, Faculty of Veterinary Medicine, Universitas Airlangga, Surabaya, East Java, Indonesia; 8 Department of Health, Faculty of Vocational Studies, Universitas Airlangga, Surabaya, East Java, Indonesia; 9 Caprifarmindo Laboratories, Bandung, West Java, Indonesia

**Keywords:** binary ethylenimine, cytopathic effect, foot-and-mouth disease virus GR12, foot-and-mouth disease, formaldehyde, Indonesia, vaccine development

## Abstract

**Background and Aim:**

Foot-and-mouth disease (FMD) is a highly contagious transboundary livestock disease that poses serious economic and food security threats. In Indonesia, recurrent outbreaks since 2022 have highlighted the urgent need for localized vaccines to ensure sustainable control. Inactivation is a critical step in the development of inactivated FMD vaccines. While formaldehyde (FA) and binary ethylenimine (BEI) have been used individually or in combination for virus inactivation, their efficacy against new outbreak strains requires reevaluation. This study aimed to determine the optimal FA-BEI concentration and incubation time for inactivating the FMD virus (FMDV) Gresik sample no.12 (GR12) strain, isolated during the third outbreak in Gresik, East Java.

**Materials and Methods:**

FMDV serotype O GR12 was propagated in baby hamster kidney (BHK-21) cells, with titers determined by tissue culture infectious dose (TCID_50_). Four FA-BEI formulations were evaluated: F1 (0.04% FA + 2 mM BEI), F2 (0.1% FA + 1 mM BEI), F3 (0.1% FA + 2 mM BEI), and F4 (0.2% FA + 1 mM BEI). Inactivation was conducted at 37°C with sampling at 24, 48, and 72 h. Validation was performed through three sequential blind passages on BHK-21 monolayers, and cytopathic effects (CPEs) were scored and statistically analyzed.

**Results:**

FMDV GR12 propagated successfully in BHK-21 cells with titers of approximately 1.9 × 10^8^ TCID_50_/mL. All FA-BEI combinations reduced CPE formation at 48 and 72 h; however, only F4 (0.2% FA + 1 mM BEI) achieved complete inactivation, showing no CPE across all passages after 72 h. Increasing BEI concentration alone did not significantly enhance inactivation. Statistical analysis confirmed that F4 was significantly more effective (p < 0.05) than other formulations.

**Conclusion:**

The combination of 0.2% FA and 1 mM BEI at 37°C for 72 h effectively inactivated FMDV GR12, establishing a baseline protocol for strain-specific inactivation in Indonesia. This study underscores the necessity of tailoring inactivation strategies to emerging FMDV strains and provides a practical foundation for localized vaccine production. Limitations include reliance solely on CPE validation; future studies should assess antigenic integrity and immunogenicity of inactivated viral proteins to ensure vaccine efficacy.

## INTRODUCTION

Foot-and-mouth disease (FMD) is a highly contagious vesicular disease that primarily affects cloven-hoofed animals such as cattle and pigs, leading to severe economic losses in the livestock industry [1]. The causative agent, FMD virus (FMDV), belongs to the genus *Aphthovirus* within the family *Picornaviridae*. FMDV carries a positive-sense, single-stranded RNA genome that is translated into a polyprotein, subsequently cleaved into structural and non-structural proteins. The extensive genetic and antigenic diversity among FMDV strains complicates vaccine development and control measures, as immune responses elicited by one strain often provide limited cross-protection against others [2]. Consequently, region-specific vaccines must be maintained to achieve effective control [3].

In Indonesia, FMD re-emerged in 2022 after more than a century, with the last recorded outbreak occurring in 1887 [4]. To curb the spread, the government implemented a nationwide vaccination program for susceptible ungulates [5]. Despite these efforts, outbreaks reoccurred in 2023 and 2024, marking the second and third waves of infection [6, 7]. A major challenge is that most vaccines used in Indonesia are imported. To strengthen national preparedness and reduce dependency on imports, local vaccine development efforts have focused on candidate strains isolated during the first outbreak [8, 9].

Inactivation represents one of the most critical stages in producing inactivated FMD vaccines. The choice of inactivation agents significantly influences vaccine safety and effectiveness, as incomplete virus inactivation can result in post-vaccination outbreaks [10, 11].Despite decades of global use of inactivated FMD vaccines, the effectiveness of virus inactivation protocols remains a persistent challenge, especially when addressing newly emerging outbreak strains. Binary ethylenimine (BEI), the primary inactivation agent since the 1980s, offers advantages in preserving antigenic structures but often exhibits a low inactivation rate when used alone [12, 13]. Conversely, formaldehyde (FA), once considered a standard inactivation agent, has been shown to cause incomplete inactivation and undesirable modifications to viral antigenicity [14, 15]. To overcome these limitations, FA and BEI are often combined [16]; however, most of the available evidence is based on older strains or experimental conditions that may not reflect the evolving genetic diversity of contemporary FMDV isolates. In Indonesia, FMD re-emerged in 2022 after more than a century, and subsequent outbreaks in 2023 and 2024 suggest that viral adaptation and strain variation may influence the success of standard inactivation protocols. Current knowledge about the inactivation kinetics of Indonesian outbreak strains, particularly those from the third outbreak (Gresik sample no.12 [GR12] isolate), remains limited. Importantly, while a combination of 0.04% FA and 1 mM BEI successfully inactivated the first outbreak strain [8], it is unknown whether these parameters are sufficient for more recent isolates, raising concerns about vaccine safety and immunogenic fidelity if outdated protocols are applied directly.

This study aimed to evaluate the effectiveness of different FA and BEI combinations, along with varying incubation times, for inactivating the FMDV serotype O GR12 strain isolated during Indonesia’s third outbreak. Specifically, the research sought to identify the optimal concentration and incubation duration required to achieve complete viral inactivation while minimizing cytopathic effects (CPE) in baby hamster kidney (BHK-21) cells. By systematically comparing four FA-BEI formulations, this study provides empirical data on strain-specific inactivation requirements and establishes a protocol tailored for localized vaccine production. The findings are expected to inform future FMD vaccine development in Indonesia and other Southeast Asian countries, where reliance on imported vaccines continues to hinder sustainable outbreak management.

## MATERIALS AND METHODS

### Ethical approval

This study was conducted entirely *in vitro*. All experimental procedures were reviewed and approved by the Animal Care and Use Committee of the Faculty of Veterinary Medicine, Universitas Airlangga, Surabaya, Indonesia (Approval No. 1.KEH.096.07.2024).

### Study period and location

The study was conducted between December 2024 and March 2025 at the Research Center for Vaccine Technology and Development (RCVTD), Institute of Tropical Diseases, Universitas Airlangga, Surabaya, Indonesia.

### Study design

The study employed a randomized controlled trial design. Data were obtained by comparing treatment groups with a control group [17]. Four treatment groups were prepared using varying concentrations of FA and BEI:

- F1: 0.04% FA + 2 mM BEI
- F2: 0.1% FA + 1 mM BEI
- F3: 0.1% FA + 2 mM BEI
- F4: 0.2% FA + 1 mM BEI.

**Figure 1:**
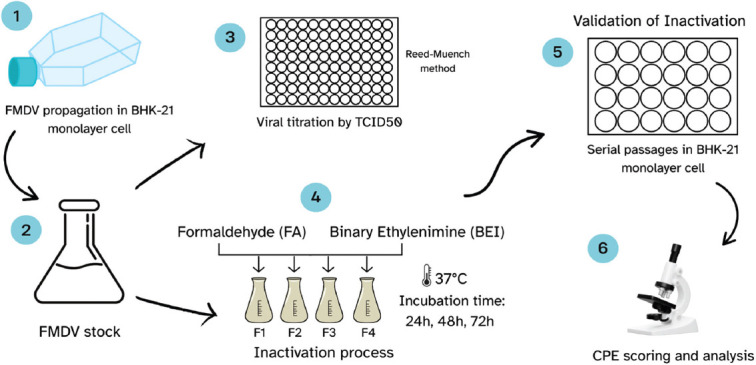
Workflow of the foot-and-mouth disease virus inactivation process.

A positive control consisting of untreated virus suspension was also included. The overall inactivation workflow is illustrated in Figure 1.

### Virus isolation and characterization

The FMDV serotype O used in this study was obtained from the viral collection of RCVTD, originally isolated from naturally infected cattle during the third FMD outbreak in Gresik Regency, East Java, Indonesia. The isolate was designated GR12 (Gresik, sample 12). The virus identity was confirmed using reverse transcription polymerase chain reaction (PCR), and viral stocks were harvested at the ninth passage.

### Cell culture and viral propagation

The GR12 isolate was propagated in BHK-21 monolayer cells at approximately 80% confluency following 24–48 h of incubation. Cells were maintained in minimum essential medium (MEM; Gibco, USA) supplemented with 10% newborn calf serum (NCS) (NCS; Gibco^™^, New Zealand). BHK-21 cells were seeded into T25 flasks (NEST^®^, China) at 7.5 × 10^4^ cells per flask. After discarding the culture medium and washing once with Phosphate-Buffered Saline (PBS), 1 mL of virus suspension was inoculated. Cultures were incubated at 37°C with 5% CO_2_, with gentle tilting every 15 min for 1 h to ensure even distribution of the virus [18]. An inoculation medium containing MEM with 5% NCS was added, and cells were further incubated. CPE was monitored daily for 24–72 h using an inverted microscope (Nikon TMS, Japan) at 100× magnification. Viral supernatants were harvested when CPE exceeded 80%, and the virus was passaged 3 times before titration.

### Viral titration

BHK-21 cells were seeded into 96-well plates (NEST, China) at a density of 1 × 10^4^ cells per well. Ten-fold serial dilutions (10^−1^–10^−10^) of viral suspension were prepared, with MEM + 5% NCS as the negative control and undiluted virus as the positive control. Each dilution was tested in eight replicates. Following 1 h of incubation, 100 μL of MEM overlay was added, and plates were incubated at 37°C with 5% CO_2_ for 48 h. CPE was evaluated daily [19]. After 48 h, cells were washed with 1× Dulbecco’s PBS, stained with 0.4% crystal violet, and air-dried at 27°C. Wells with CPE were recorded, and 50% tissue culture infectious dose (TCID_50_) values were calculated using the following formula [20–22].

The proportionate distance (PD) was calculated as:


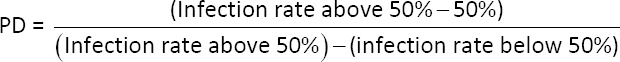


Calculation of TCID_50_/0.1 mL:

logID_50_ = log (dilution with [50 with [50% positive]) + PD × (– log [dilution factor]).

### Preparation of inactivating agents

BEI was prepared as described by Sarachai *et al*. [23]. Briefly, 0.041 g of 2-bromoethylamine (BEA) hydrobromide (Sigma-Aldrich, Switzerland) was dissolved in 2 mL of 0.175 N NaOH (Merck, Germany) to yield a 0.1 M solution. The solution was incubated at 37°C for 1 h, during which pH decreased from 12 to 8.5, confirming conversion of BEA to BEI, monitored with a pH meter (SevenEasy, Mettler Toledo, Switzerland). Solutions were freshly prepared before use.

All BEI preparation and inactivation procedures were performed in a biosafety cabinet level 2 with full PPE. Following inactivation, BEI was neutralized to a final concentration of 2% (v/v) using sodium thiosulfate (Sigma-Aldrich, USA) [24].

### Virus inactivation procedure

Based on protocols used for Indonesia’s first FMD outbreak [8], 50 mL of GR12 supernatant was treated with four FA-BEI formulations. FA solutions were prepared from 37% absolute FA and BEI from the freshly prepared stock. Virus suspensions were incubated in Erlenmeyer flasks on a Thermo Scientific Cimarec+ Stirring Hotplate (Thermo Scientific, USA) at 37°C with agitation at 3 × *g*. BEI solutions were added dropwise, followed by FA to achieve target concentrations (0.04%–0.2%). Incubation continued for 72 h, with samples collected at 24, 48, and 72 h for validation [25].

### Validation of FMDV inactivation

To verify inactivation and confirm the absence of residual infectivity, three blind serial passages were performed on BHK-21 monolayers, each with a 72 h incubation. Each formulation was tested in six replicates [26]. BHK-21 cells were seeded in 24-well plates (NEST, China) until they became confluent, then inoculated with 50 μL of inactivated virus or the positive control. After 1 h of adsorption at 37°C in 5% CO_2_, inocula were removed and replaced with 500 μL of MEM + 5% NCS. Cells were monitored daily for CPE.

CPE severity was graded on a 0–4 scale [27]:

- 0: No CPE
- 1: CPE in 0%–25% of cells
- 2: CPE in >25%–50% of cells
- 3: CPE in >50%–75% of cells
- 4: CPE in >75%–100% of cells.

Observed CPE included membrane disorganization, cell clumping, rounding, breakdown of extracellular bridges, and detachment [28, 29]. Non-inactivated virus (positive control) was expected to replicate consistently with stable CPE across passages, whereas successful inactivation would result in progressive reduction and eventual absence of CPE.

### Statistical analysis

Data were expressed as mean ± standard deviation (SD). Bar graphs and line charts were generated using Microsoft Excel 2019 (Microsoft Corp., USA). Statistical analysis of CPE scores was performed using Kruskal-Wallis and Mann-Whitney tests in the Statistical Package for the Social Sciences version 27 (IBM Corp., NY, USA). Differences were considered statistically significant at p < 0.05.

## RESULTS

### Virus isolation and propagation

**Figure 2:**
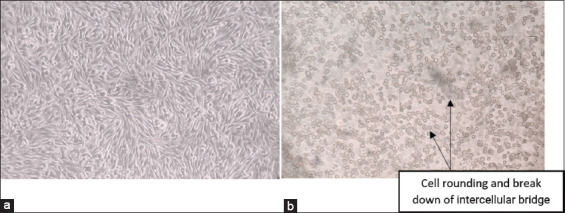
Appearance of baby hamster kidney (BHK-21) cells. (a) Normal elongated and tapered morphology and (b) BHK-21 cells infected with FMDV showing CPE. Rounding, disruption of intercellular junctions, cell death, and detachment from the flask surface. Images were captured using an inverted microscope at 100× magnification. CPE = Cytopathic effects, FMDV = Foot-and-mouth disease virus.

FMDV GR12 was produced in BHK-21 monolayer cells with 10% NCS. After 24 h of incubation at 37°C with 5% CO_2_, the cells exhibited FMDV-specific CPE, characterized by cell rounding, breakdown of intercellular bridges, and cell death (Figure 2) [28, 29]. The appearance of CPE indicated that FMDV successfully infected and replicated in BHK-21 cells. Within 24 h, GR12 was able to induce CPE of up to 85% in BHK-21 cells. The cell culture supernatant was then harvested, and the TCID_50_ assay was carried out.

### Virus titration

In a 96-well plate, GR12 was diluted from 10^1^ to 10^10^ in eight replicates. After staining with crystal violet, the unstained and stained areas were observed. Any unstained, empty area on a blue-stained cell background indicated the presence of viral infection. The absence of any empty white spots across the blue-stained background was considered indicative of a clear absence of viral infection [30]. The GR12 titer was determined using the Reed and Muench [20] method, and the calculation results are shown in Table 1. After calculation, viral titers were approximately log 10^8.28^ or 1.9 × 10^8^ TCID_50_/mL for GR12 before inactivation.

**Table 1:** Tissue culture infectious dose_50_ calculation.

Viral dilution	CPE formation		Accumulated numbers		Total (A + B)	Percentage infection A/(A + B) × 100%
	
	Number of infected wells (CPE+)	Number of uninfected wells (CPE−)	Infected (CPE+) (A)	Uninfected (CPE−) (B)		
10^−1^	8	0	54	0	54	100
10^−2^	8	0	46	0	46	100
10^−3^	8	0	38	0	38	100
10^−4^	7	1	30	1	31	97.7
10^−5^	6	2	23	3	26	88.5
10^−6^	6	2	17	5	22	77.3
10^−7^	5	3	11	8	19	57.9
10^−8^	4	4	6	12	18	30
10^−9^	2	6	2	18	20	10
10^−10^	0	8	0	26	26	0

CPE = Cytopathic effects

### FMDV inactivation using FA and BEI

In this study, FMDV serotype O, isolated from the third outbreak, was exposed to different concentrations of FA and BEI. Each formula was incubated for 72 h, and the mixture was sampled at 24 h, 48 h, and 72 h to validate inactivation by CPE analysis. The absence of viral replication (−) or CPE was assessed by microscopic inspection [31]. The CPE on BHK-21 cells was measured for each inactivated formula (F1–F4). Scores ranged from 0 (no CPE) to 4 (severe CPE, >75%–100%) [27].

### CPE observations

**Figure 3:**
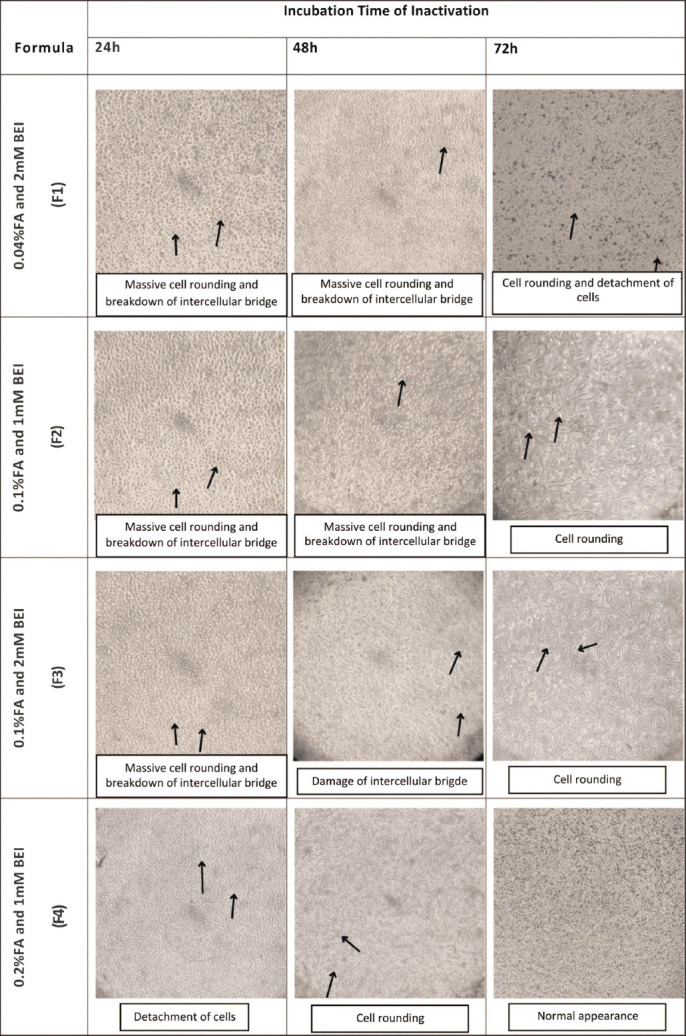
Cytopathic effects observation between treatment groups (F1-F4) under an inverted microscope at 100× magnification.

CPE formation after inoculation is shown in Figure 3.

- CPE score 4 (>75%–100% CPE) appeared in F1–F4 at 24 h and in F1 and F2 at 48 h. At this stage, all cells were rounded, extracellular bridges were damaged, and cells detached from the plate surface
- CPE score 3 (>50%–75% CPE) was observed in F3 at 48 h, with most cells rounded, extracellular bridges damaged, and half of the cells detached
- CPE score 2 (>25%–50% CPE) was observed in F4 at 48 h, where cells were moderately rounded, extracellular bridges began to break down, and some cells detached
- CPE score 1 (0%–25% CPE) was observed in F1–F3 at 72 h, with mild rounding and intact extracellular bridges
- CPE score 0 (no CPE) was observed in F4 at 48 h, with normal cell morphology and intact extracellular bridges.

**Figure 4:**
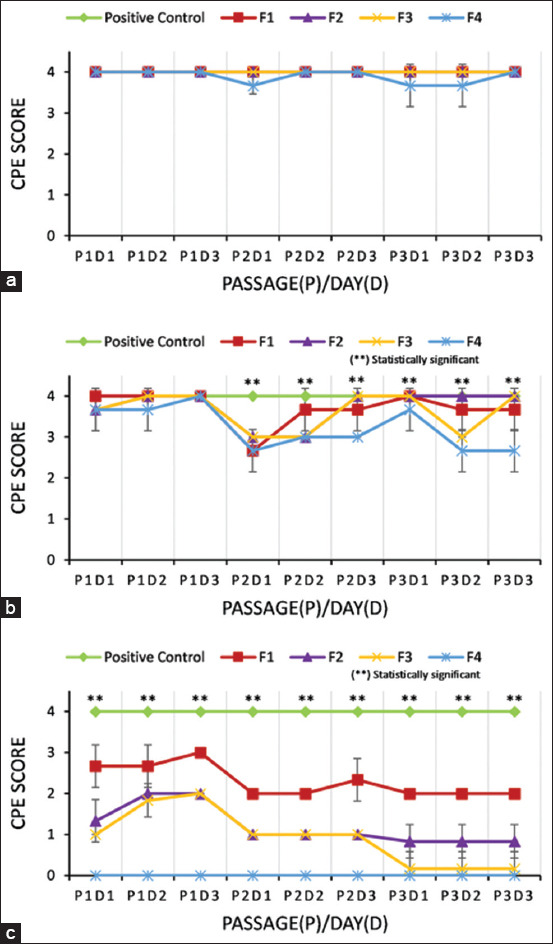
CPE score during three passages for (a) 24 h incubation time of inactivation, (b) 48 h incubation time of inactivation, and (c) 72 h incubation time of inactivation. The mean ± standard deviation of replicates was used for each formula. F1: 0.04% FA and 2 mM BEI; F2: 0.1% FA and 1 mM BEI; F3: 0.1% FA and 2 mM BEI; and F4: 0.2% FA and 1 mM BEI. The significant difference (p > 0.05) between formulas in three passages (P1-P3) is indicated by asterisks (**) after 48 and 72 h incubation of the inactivation experiment. CPE = Cytopathic effects, FA = Formaldehyde, BEI = Binary ethylenimine.

The CPE score was analyzed and presented as mean ± SD (Figure 4). Observation at 24 h showed a score of 4 for nearly all formulations in all passages. Only F4 showed a CPE score below 4 on three occasions: Day 1 of passage 2, and days 1 and 2 of passage 3. This indicates that inactivation had no effect on CPE formation, which was consistent with statistical analysis (p > 0.05).

At 48 h, all formulas showed a decrease in CPE scores across passages, with significant differences (p < 0.05) observed from passage 2 to passage 3. At 72 h, a significant difference (p < 0.05) was observed for all formulations across all passages.

### Comparative effectiveness of FA-BEI formulations

At 48 h, a significant difference was observed between NC and F1, F2, and F3 in the 2^nd^ passage on day 1, but no significant difference was noted in the 3^rd^ passage on day 3 (p > 0.05) (Figure 4). This indicated that the virus was still replicating and forming CPE. By contrast, NC and F4 differed significantly in both the 2^nd^ and 3^rd^ passages on all days (p < 0.05). This showed that F4 was more capable of inactivating the virus than the other formulations, significantly inhibiting CPE formation after 48 h of incubation.

After 72 h of inactivation, a significant difference was observed between NC and all formulas (F1–F4) across all passages and days. This indicates that a 72-h incubation period can significantly reduce CPE formation in all formulas.

**Figure 5:**
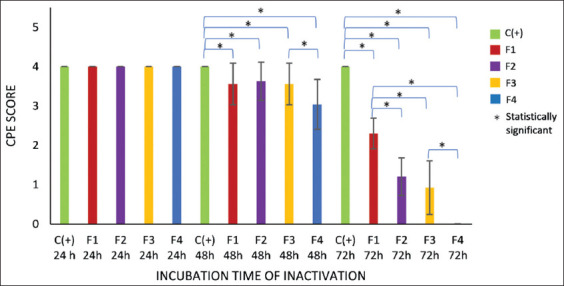
Effectiveness of FA and BEI in inactivating FMDV Gresik sample no.12 after incubation for 24 h, 48 h, and 72 h. Inactivation of FMDV with F1: 0.04% FA and 2 mM BEI (red bars); F2: 0.1% FA and 1 mM BEI (purple bars); F3: 0.1% FA and 2 mM BEI (yellow bars); and F4: 0.2% FA and 1 mM BEI (blue bars); green bars represent the positive control (C+, virus suspension without inactivation). Asterisks indicate significant differences (p > 0.05) between the two groups (*). FA = Formaldehyde, BEI = Binary ethylenimine, FMDV = Foot-and-mouth disease virus.

Key findings include:

- All formulas significantly reduced CPE formation at 48 and 72 h (Figure 5).
- No significant difference was observed between F1, F2, and F3 at 48 h.
- No significant difference was observed between F2 and F3 at 72 h (p > 0.05), indicating similar inactivation levels.
- A significant difference was observed between F3 and F4 after both 48 and 72 h.
- The positive control (virus without inactivation) consistently showed >75% CPE, confirming active viral replication.
- Complete inactivation (CPE = 0 across all passages) occurred only with F4 (0.2% FA + 1 mM BEI at 72 h).

## DISCUSSION

### Virus propagation and titration

BHK-21 is a hamster-derived cell line that is commonly used for FMD vaccine production [32]. Within 24 h of incubation, FMDV-specific CPE appeared in BHK-21 cells, characterized by rounding, intercellular bridge breakdown, and cell death, marked by cell detachment from the flask surface, as demonstrated in this study. These findings are consistent with previous reports of FMDV infection in cell culture [33, 34]. In Tobing *et al*. [9], cell detachment occurred within 72 h of incubation using the first Indonesian FMD outbreak isolate, whereas in this study, detachment occurred within 24 h, indicating a more rapid replication rate.

Based on TCID_50_ calculations, the FMDV titer was approximately 1.9 × 10^8^/mL. By comparison, the first Indonesian outbreak isolate had a TCID_50_ value of 10^9^/mL. This finding is consistent with other studies, where FMDV vaccine antigens are produced at titers around 10^8^ TCID_50_/mL [35]. The TCID_50_ required for FMD vaccine production is generally above 10^6^ TCID_50_/0.1 mL [36].

### Inactivation validation

Successful pathogen inactivation requires careful consideration of pathogen type (virus, bacteria, spores, or prions), sample characteristics (organic content, volume, and concentration), inactivation method (chemical, autoclaving, and irradiation), and environmental conditions (temperature, humidity, airflow, pH, and contact time). High-containment laboratories commonly employ combinations of chemical, heat, and radiation treatments [37].

In this study, we investigated the inactivation kinetics of serotype O isolate GR12, an emergent strain from Indonesia’s third FMD outbreak with unknown responsiveness to dual-agent treatment.

FA remains one of the most widely used inactivating agents for FMDV [13, 15]. It crosslinks nucleic acids and proteins, stabilizing viral particles but potentially modifying antigenicity [38]. BEI, in contrast, primarily interacts with nucleic acids through intercalation, causing less protein crosslinking and preserving viral antigenicity [39–41]. The combined use of FA and BEI provides a synergistic effect: FA stabilizes viral proteins, while BEI ensures complete genetic inactivation [38, 42]. Indeed, prior experiments showed that FA-BEI combinations enhanced immune responses to SAT serotype FMDV [41].

BEI is typically used at 1–3 mM concentrations with treatment times ranging from several hours to 30 h at 26–37°C [43]. FA concentrations between 0.01% and 0.2% are commonly applied for 48–96 h at 37°C [8, 9, 11, 44]. This study advances inactivation methodology by optimizing FA-BEI combinations to balance complete viral inactivation with antigenic preservation, a critical step for local vaccine production.

Continuous passaging of treated viral suspensions on uninfected target cells is an established standard for confirming complete inactivation [31]. The presence or absence of CPE provides direct validation: Untreated samples maintain CPE, while successfully inactivated samples do not [30]. Here, three blind passages were performed for each inactivation formula, each incubated for 3 days.

- F1 (0.04% FA + 1 mM BEI) and F2 (0.1% FA + 1 mM BEI): CPE was observed at 24, 48, and 72 h
- F3 (0.1% FA + 2 mM BEI): CPE was eliminated only at passage three, but still present in passages one and two
- F4 (0.2% FA + 1 mM BEI): No CPE was observed after 72 h, confirming complete inactivation.

Thus, the absence of CPE with F4 validated that the virus was inactive and unable to infect host cells.

### Comparison with previous studies

Previous study by Kurniawan *et al*. [8] on the first Indonesian FMDV outbreak strain reported complete inactivation with 0.04% FA and 1 mM BEI. However, this combination failed to inactivate the GR12 strain from the third outbreak. In addition, a study by Tobing *et al*. [9] confirmed that 0.2% FA was sufficient to prevent detectable CPE in the first outbreak isolate. These findings suggest that the GR12 strain may have reduced sensitivity to earlier inactivation protocols, likely due to viral adaptation across outbreaks, necessitating updated standards.

Temperature and incubation time are critical for successful inactivation [45–47]. As demonstrated here, higher temperatures and longer exposure (72 h at 37°C) ensured complete inactivation. Increasing BEI concentration from 1 to 2 mM did not significantly enhance inactivation, differing from reports that found BEI concentration effects only at low temperatures (4°C) [48]. Residual virus titers were shown to correlate proportionally with contact time, BEI concentration, and temperature.

Tobing *et al*. [9] also reported that FA-inactivated FMDV initially induced transient CPE, but subsequent passages restored normal cell morphology and growth. Similarly, in this study, early passages showed higher CPE that progressively decreased by passage three, with no detectable CPE even in F3 at the third passage.

### Implications for vaccine development

In resource-limited countries, access to high-quality vaccines is restricted, with reliance on international donors [49]. Developing locally produced vaccines is therefore a strategic priority for improving outbreak control and livestock productivity. This study provides foundational data for establishing a strain-specific inactivation protocol for Indonesia, enabling a shift toward localized vaccine manufacturing and rapid outbreak response.

Moreover, these findings offer valuable insights for Southeast Asian countries where serotype O is prevalent. The optimized FA-BEI protocol developed here represents a scalable, region-specific inactivation approach, bridging laboratory-scale validation with potential for commercial vaccine production.

### Limitations

Validating inactivation is critical for confirming that inactivated samples are safe and do not pose a risk of infection. While CPE observation remains a useful method for assessing virus inactivation, it should not be the sole approach. Molecular assays, such as quantitative PCR (qPCR), can complement CPE analysis to provide a more comprehensive validation, particularly in cases where CPE are absent [30, 35, 50].

Another limitation relates to the stability and immunogenicity of the vaccine. Prolonged inactivation or the use of improper concentrations of inactivating agents may compromise structural stability and reduce the immunogenic potential of the vaccine [43, 51].

Future studies can build on this optimized FA-BEI protocol by further assessing structural protein integrity and immunogenic retention. Such evaluations will be essential for advancing the development of next-generation FMD vaccines that maintain superior antigenic fidelity.

## CONCLUSION

This study successfully optimized a dual-agent inactivation protocol using 0.2% FA and 1 mM BEI for 72 h, which completely prevented the formation of CPE in the Indonesian GR12 strain from the third FMD outbreak. Compared with earlier strains, which were fully inactivated with milder conditions (0.04% FA + 1 mM BEI), the GR12 strain demonstrated reduced sensitivity to standard protocols, suggesting adaptive viral changes across outbreaks. These findings provide critical evidence that strain-specific inactivation kinetics must be re-evaluated during successive outbreaks to ensure effective vaccine development.

The optimized inactivation strategy offers practical applicability for local vaccine production, reducing reliance on imported vaccines and strengthening Indonesia’s ability to respond rapidly to FMD emergencies. Moreover, this protocol provides a scalable framework that can be adapted for other Southeast Asian countries where serotype O FMDV is endemic, supporting region-wide vaccination policies and livestock protection.

The strength of this study lies in systematically comparing different FA-BEI concentrations, confirming complete inactivation across multiple passages, and highlighting the importance of antigenic preservation for vaccine efficacy. However, validation relied solely on CPE analysis; future studies should integrate molecular assays (qPCR) and immunogenicity testing to ensure comprehensive confirmation of viral inactivation and retention of protective epitopes.

Looking forward, further research should assess structural protein integrity, antigenic fidelity, and immune response profiles induced by FA-BEI inactivated vaccines. Such studies will pave the way for the development of next-generation FMD vaccines that are not only strain-specific but also more immunogenically stable and protective.

This study provides foundational data for establishing a region-specific inactivation protocol, marking an essential step toward Indonesia’s transition to localized FMD vaccine manufacturing. By tailoring inactivation approaches to emerging viral strains, this work advances both scientific knowledge and practical disease control strategies, ensuring sustainable livestock production and improved outbreak preparedness.

## AUTHORS’ CONTRIBUTIONS

FAR: Conceptualized and validated the manuscript. FAR, JR, and SK: Methodology. FKM, NSS, ZD, and DUF: Laboratory work and formal analysis. FKM: Writing – original draft preparation. FKM, NSS, and FAR: Writing – review and editing. DD, YP, HS, and MM: Supervised the manuscript. All authors have read and approved the final manuscript.
